# Beak and feather disease virus in wild and captive parrots: an analysis of geographic and taxonomic distribution and methodological trends

**DOI:** 10.1007/s00705-016-2871-2

**Published:** 2016-05-05

**Authors:** Deborah J. Fogell, Rowan O. Martin, Jim J. Groombridge

**Affiliations:** Durrell Institute of Conservation and Ecology, University of Kent, Canterbury, CT2 7NZ UK; World Parrot Trust, Glanmor House, Hayle, Cornwall TR27 4HB UK; Percy FitzPatrick Institute of African Ornithology, DST/NRF Centre of Excellence, University of Cape Town, Cape Town, South Africa

## Abstract

Psittacine beak and feather disease (PBFD) has emerged in recent years as a major threat to wild parrot populations and is an increasing concern to aviculturists and managers of captive populations. Pathological and serological tests for screening for the presence of beak and feather disease virus (BFDV) are a critical component of efforts to manage the disease and of epidemiological studies. Since the disease was first reported in the mid-1970s, screening for BFDV has been conducted in numerous wild and captive populations. However, at present, there is no current and readily accessible synthesis of screening efforts and their results. Here, we consolidate information collected from 83 PBFD- and BFDV-based publications on the primary screening methods being used and identify important knowledge gaps regarding potential global disease hotspots. We present trends in research intensity in this field and critically discuss advances in screening techniques and their applications to both aviculture and to the management of threatened wild populations. Finally, we provide an overview of estimates of BFDV prevalence in captive and wild flocks alongside a complete list of all psittacine species in which the virus has been confirmed. Our evaluation highlights the need for standardised diagnostic tests and more emphasis on studies of wild populations, particularly in view of the intrinsic connection between global trade in companion birds and the spread of novel BFDV strains into wild populations. Increased emphasis should be placed on the screening of captive and wild parrot populations within their countries of origin across the Americas, Africa and Asia.

## Introduction

Pathogens responsible for emerging infectious diseases (EIDs) have become a major concern in conservation biology owing to their potential for rapid evolution and the effect that an epidemic may have on vulnerable species [1]. Consequently, understanding infectious diseases and their management in wildlife populations has become increasingly important to conservationists [2]. Assessing the prevalence and impact of disease can be challenging, particularly during the outbreak of a novel pathogen [3]. Data collected and used in these circumstances often vary in the sampling or assessment method used, frequently with imperfect diagnostic tests providing the only available insight into infection incidence within a population [4, 5]. Consequently, synthesising multiple sources of information across many species can provide insight into how to improve management of infectious disease, identify knowledge gaps, and reveal where improvements in surveillance methods might be required.

Psittacine beak and feather disease (PBFD) has been detected in both wild and captive parrot populations since the mid-1970s. The disease has been found to be widely infectious and often fatal, affecting both Old and New World psittacine species. PBFD is thought to have been first documented in the late 1880s in wild Australian *Psephotus* parrots and was described as feathering abnormalities that impaired their flight [6]. Most commonly affecting immature and fledgling birds, classical symptoms include symmetrical loss of contour, tail and down feathers and subsequent replacement by dystrophic and necrotic feathers that fail to grow soon after emergence from the follicle [7–9]. Beak deformities such as fractures, abnormal elongation and palatine necrosis are also typical symptoms of PBFD, but their presence and severity vary from species to species [10]. Other clinical symptoms include lethargy, depression, diarrhoea and immunosuppression, which are individually variable, sometimes lead to death, and may depend on the virulence of the viral strain or the route of viral exposure [11].

Beak and feather disease virus (BFDV) is a member of the family *Circoviridae* [12], which includes the smallest known autonomously replicating pathogenic animal viruses [13–15]. The first complete BFDV genome sequence confirmed its relationship to other circoviruses [16]. The structure of BFDV isolated from viral inclusion bodies was determined to be a non-enveloped, icosahedral virion between 14 and 16 nm in size and containing a single-stranded DNA genome approximately 1.7 to 2.0 kilobases in length [10].

Until the early 1990s, histology and recovery of virions were the primary means of determining whether a bird was infected with BFDV. The first haemagglutination (HA) and haemagglutination inhibition (HI) assays were then developed as a technique for both the identification and quantification of virus recovered from BFDV-positive birds [17]. Since the initial description of the syndrome, several attempts have been made to culture the virus *in vitro* in order to provide a source of antigen for vaccinations, but these have not yet been successful [16, 18, 19]. The lack of an effective vaccine has compelled researchers to develop techniques to further examine the molecular genetics of the virus; encouraging development of oligonucleotide-probe-based methodologies such as dot-blot DNA hybridization, DNA *in situ* hybridization, and a polymerase chain reaction (PCR)-based assay [20, 21]. Critically, as infection and the presentation of clinical disease are fundamentally different [22], these techniques provided a means to determine whether infection was present, even when the individual being studied was asymptomatic. The small size of circoviruses means that whole-genome sequencing is relatively quick and inexpensive, facilitating investigations of phylogenetic relationships between different viruses within the family and between strains of the same virus occurring in different hosts or global regions [23–25].

PBFD has become a major cause for concern to conservationists and aviculturists as the disease has spread rapidly across the world due to BFDV’s high environmental persistence and ability to shift between closely related host species [26–28]. BFDV is readily transmitted through contact with contaminated feather dust, surfaces or objects [29], and it can also be passed directly from a female to her offspring [10, 30]. The management of PBFD in captivity is economically important in some countries; for example, it was estimated that aviculturists in South Africa lose up to 20 % of their flock to the disease annually [31]. Worryingly, many wild populations of vulnerable species are also affected, including the Cape parrot (*Poicephalus robustus*) of South Africa [25], the Australian orange-bellied parrot (*Neophema chrysogaster*) [28, 32] and swift parrot (*Lathamus discolor*) [33], and the Mauritius (or “echo”) parakeet (*Psittacula echo*) [30]. Therefore, understanding the mechanics behind the spread of BFDV and how to test for its prevalence has taken on a renewed global relevance.

Concern over the implications for conservation, aviculture and biosecurity together with methodological advances in the detection of the virus has prompted a recent increase in research effort. The development of new methodologies has provided the basis on which researchers are now able to model the potential routes of transmission around the world [34], link BFDV prevalence to management-related tools for endangered species recovery [35], and determine the ways in which anthropogenic activities have changed the way in which the virus is evolving due to recombination [36]. Remarkably, whilst there are many research teams worldwide working on BFDV and PBFD, there is a severe lack of synthesised knowledge on the primary screening methods being used, the species affected, and, consequently, potential disease hotspots that have lacked attention. Here, we aim to consolidate the most pertinent patterns and methods emerging from the literature published since the first scientific description of PBFD in 1984 to provide both a qualitative and quantitative overview of approaches and screening results. Our review provides a much-needed source of information for use by conservation practitioners regarding BFDV prevalence estimates in captive and wild flocks. Our objective is not to provide an exhaustive description of each technique, but instead to analyse the trends in how screening has progressed over the last three decades and provide an overview of prevalence estimates for this infectious disease alongside broader implications for biosecurity and conservation.

## Methods

### Literature search

Searches for literature were conducted by entering English key words and terms into Google Scholar and were selected to balance search sensitivity with specificity. The terms were “Beak and feather disease virus”, “Psittacine beak and feather disease”, “Beak and feather disease”, “Psittacine circovirus”, “BFDV screening”, “PBFD screening”, “BFDV detection” and “PBFD detection”. Acquisition of literature was restricted to only those articles that had been published in academic journals or as conference proceedings up to and including July 2015, thus excluding any theses and organisational reports.

### Analysis

Information extracted from each publication included the year published, whether the birds studied originated in the wild or in captivity, the host species, the country of origin of all specimens, tissue types and laboratory methods used in the detection of BFDV, and the outcome of diagnostic tests, including detection prevalence. If an estimate of total population prevalence was provided, this value was also recorded.

The publications were grouped into five-year intervals to examine the trend in the number of publications produced over time. If multiple species from the same country of origin were involved in the same study, the country of origin was recorded once per publication. If the study was based on captive individuals and a different country of origin for a specimen was not otherwise clearly stated in the publication, it was assumed that the country in which the study was undertaken was the country of origin. In multiple instances, the countries in which the tests were conducted differed from the country of origin of the parrots. In such instances, it is not possible to determine if the parrots were infected with the virus in the country of origin or upon arrival at their destination. Thus, the presence of the virus in a parrot originating from a given country does not necessarily indicate its presence in wild or captive populations in the country of origin. Where a study used specimens from both captive and wild individuals from the same country, the country of origin for each specimen was recorded once per category for each publication. For example: Regnard *et al.* [37] screened specimens from both captive and wild populations of *Poicephalus robustus*, and this information was recorded by adding South Africa once to each category. Maps were produced using ArcGIS 10.2.1 [38], displaying the results of captive and wild specimens independently. Seven publications did not declare whether the specimens obtained were of wild or captive origin. These reports pertained to five incidences from Australia, one from the United States of America (USA) and one from Brazil. These incidences were all excluded from the analyses of geographical patterns. The common names of species historically recorded as positive for PBFD/BFDV were aligned to current nomenclature as per the International Union for Conservation of Nature (IUCN) Red List database, alongside additional information regarding their current IUCN status and native geographic region.

Screening methods were recorded once per publication. The annual trends in the five most frequently used screening methods were assessed, along with the overall most commonly combined mixed-methods approaches. As with country of origin, tissues used for screening and diagnostics were divided into wild and captive specimens, and, where a study used a certain tissue type from both captive and wild individuals, that type was recorded once per category for each publication.

## Results

### Publication trends and affected species

**Fig. 1:**
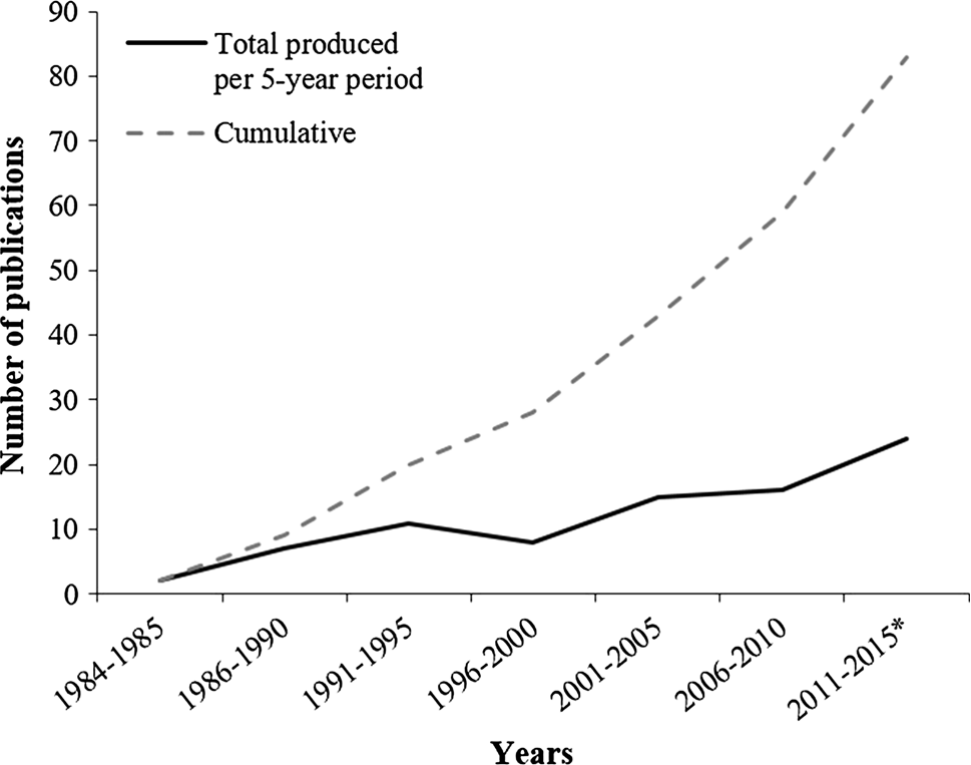
The number of publications in academic journals reporting the presence of PBFD or results of BFDV screening produced between 1984 and July 2015

There has been a linear increase in the number of publications involving testing for BFDV since the first scientific description of PBFD (Fig. 1, *R*^2^ = 0.96), with the total number of screening-based publications reaching 83 by July 2015. The total number of publications on BFDV screening and prevalence is by far the highest for the most recent period (between 2011 and July 2015), being 33.3 % higher than the number of publications for the five-year period preceding it and more than 300 % higher than the first full 5-year period from 1986-1990.

**Fig. 2:**
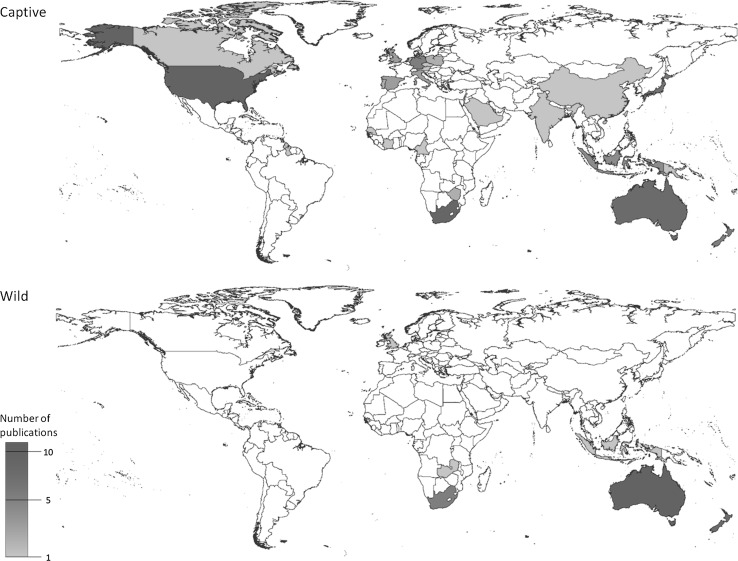
The geographical distribution of research into BFDV and PBFD in captive and wild parrots during the period 1984-July 2015. Countries are coloured according to the number of published studies involving specimens originating from that country. BFDV has been confirmed to occur in all countries from which the results of screening have been published, with the exception of Senegal. The United Kingdom is the only country in which no native parrots occur but BFDV has been detected in wild invasive flocks

Research has been focused predominantly on captive populations, encompassing 33 different countries, with the highest number of specimens originating from the USA, followed by South Africa, Australia and Japan (Fig. 2). In contrast, relatively few published studies exist for wild populations, including only eight nations. Of these studies, a substantial proportion (12 of 38) were on specimens of Australian origin. There have been no published studies of wild parrots in the New World or continental Asia. Three of these 38 studies were based on screening for BFDV among exotic introduced populations of non-native species from the United Kingdom [39], Mauritius [30] and New Zealand [40]. BFDV was reported to be present in all countries for which the results of screening of wild or captive populations have been published, with the exception of Senegal.

Of the 78 species in which BFDV has been detected in wild or captive birds (Table 1) 64.1 % (50 species) are categorised as Least Concern by the IUCN [41], 9.0 % are considered to be Near Threatened, and over a quarter are classified in Threatened categories. A declining population was observed in over 60 % of BFDV-affected host species. Of the 20 species in which BFDV has been detected among wild populations, 70.0 % (n = 14) are currently categorised as Least Concern, two are classified as Near Threatened, and the remaining four are classified in Threatened categories. Half (n = 10) were determined to have host populations increasing in population size [41]. In addition, wild populations of three subspecies have also tested positive for BFDV, all of which are native to the Oceania region.

**Table 1:** Psittacine species in which BFDV has been detected through diagnostic tests. Species for which wild populations have tested positive are marked with an asterisk (*)

Common name	Scientific name	IUCN category	Population trend	Continental region	Reference
**New World**					
Turquoise-fronted Amazon	*Amazona aestiva*	LC	Decreasing	South America	[78, 79]
White-fronted Amazon	*Amazona albifrons*	LC	Increasing	North and Central America	[59, 80]
Orange-winged Amazon	*Amazona amazonica*	LC	Decreasing	South America	[36]
Yellow-naped Amazon	*Amazona auropalliata*	VU	Decreasing	Central and South America	[20, 59]
Red-lored Amazon	*Amazona autumnalis*	LC	Decreasing	North, Central and South America	[21, 75]
Vinaceous-breasted Amazon	*Amazona vinacea*	EN	Decreasing	South America	[80]
Blue-and-yellow macaw	*Ara ararauna*	LC	Decreasing	South America	[80]
Red-and-green macaw	*Ara chloropterus*	LC	Decreasing	South America	[20]
Scarlet macaw	*Ara macao*	LC	Decreasing	South and Central America	[31, 81]
Military macaw	*Ara militaris*	VU	Decreasing	North and South America	[79]
Red-fronted macaw	*Ara rubrogenys*	EN	Decreasing	South America	[80]
Sun parakeet	*Aratinga solstitialis*	EN	Decreasing	South America	[79]
Pacific parrotlet	*Forpus coelestis*	LC	Stable	South America	[36]
Golden parakeet	*Guarouba guarouba*	VU	Decreasing	South America	[80]
Green-thighed parrot	*Pionites leucogaster*	EN	Decreasing	South America	[31, 65]
Black-headed parrot	*Pionites melanocephalus*	LC	Stable	South America	[20]
Bronze-winged parrot	*Pionus chalcopterus*	LC	Decreasing	South America	[80]
Crimson-fronted parakeet	*Psittacara finschi*	LC	Increasing	Central America	[59]
**Old World**					
Nyasa lovebird	*Agapornis lilianae*	NT	Decreasing	East Africa	[82, 83]
Black-cheeked lovebird	Agapornis nigrigenis*	VU	Decreasing	East Africa	[61, 83]
Peach-faced lovebird	*Agapornis roseicollis*	LC	Decreasing	Southern and Central Africa	[48, 84]
Australian king parrot	*Alisterus scapularis*	LC	Decreasing	Oceania	[36]
Red-winged parrot	*Aprosmictus erythropterus*	LC	Increasing	Oceania and South East Asia	[36]
Australian ringneck	Barnardius zonarius (barnardi)*	LC	Increasing	Oceania	[85, 86]
White cockatoo	*Cacatua alba*	EN	Decreasing	South East Asia	[87, 88]
Solomon’s corella	*Cacatua ducorpsii*	LC	Stable	Oceania	[21, 80]
Sulphur-crested cockatoo	Cacatua galerita*	LC	Decreasing	Oceania and South East Asia	[45, 89]
Triton cockatoo	*Cacatua galerita triton*	Not assessed		Oceania	[20, 87]
Tanimbar corella	*Cacatua goffiniana*	NT	Decreasing	South East Asia	[87, 90]
Philippine cockatoo	*Cacatua haematuropygia*	CE	Decreasing	South East Asia	[17, 90]
Major Mitchell’s cockatoo	*Cacatua leadbeateri*	LC	Stable	Oceania	[76, 84]
Moluccan cockatoo	*Cacatua moluccensis*	VU	Decreasing	South East Asia	[79, 91]
Blue-eyed cockatoo	*Cacatua ophthalmica*	VU	Decreasing	Oceania	[80]
Bare-eyed corella	Cacatua sanguinea*	LC	Increasing	Oceania and South East Asia	[92]
Yellow-crested cockatoo	*Cacatua sulphurea*	CE	Decreasing	South East Asia	[51, 91]
Citron-crested cockatoo	*Cacatua sulphurea citrinocristata*	Not assessed		South East Asia	[17, 51]
Eastern long-billed corella	Cacatua tenuirostris*	LC	Increasing	Oceania	[84, 92]
Gang gang cockatoo	Callocephalon fimbriatum*	LC	Increasing	Oceania	[26, 93]
Red-tailed black cockatoo	Calyptorhynchus banksii*	LC	Decreasing	Oceania	[93]
Glossy black cockatoo	*Calyptorhynchus lathami*	LC	Decreasing	Oceania	[93]
Vasa parrot	*Coracopsis vasa*	LC	Stable	East Africa	[79, 94]
Yellow-fronted parakeet	Cyanoramphus auriceps*	NT	Decreasing	Oceania	[46]
Red-fronted parakeet	Cyanoramphus novaezelandiae (saisseti)*	NT	Decreasing	Oceania	[52, 73]
Antipodes parakeet	*Cyanoramphus unicolor*	VU	Stable	Oceania	[42]
Eclectus parrot	*Eclectus roratus*	LC	Decreasing	Oceania and South East Asia	[20, 95]
Galah	Eolophus roseicapilla*	LC	Increasing	Oceania	[84, 92]
Red lory	Eos bornea*	LC	Decreasing	South East Asia	[96]
Horned parakeet	*Eunymphicus cornutus*	VU	Increasing	Oceania	[97]
Musk lorikeet	*Glossopsitta concinna*	LC	Stable	Oceania	[65]
Purple-crowned lorikeet	*Glossopsitta porphyrocephala*	LC	Decreasing	Oceania	[66]
Swift parrot	Lathamus discolor*	EN	Decreasing	Oceania	[33, 76]
Budgerigar	*Melopsittacus undulatus*	LC	Increasing	Oceania	[98, 99]
Orange-bellied parrot	Neophema chrysogaster*	CE	Decreasing	Oceania	[28, 100]
Kea	*Nestor notabilis*	VU	Decreasing	Oceania	[80]
Bluebonnet	*Northiella haematogaster*	LC	Decreasing	Oceania	[48, 84]
Cockatiel	*Nymphicus hollandicus*	LC	Stable	Oceania	[57, 101]
Crimson rosella	Platycercus elegans*	LC	Decreasing	Oceania	[36, 54]
Adelaide rosella	Platycercus elegans adelaidae*	Not assessed		Oceania	[54]
Yellow rosella	Platycercus elegans flaveoulus*	Not assessed		Oceania	[54]
Eastern rosella	Platycercus eximius*	LC	Increasing	Oceania	[45, 52]
Brown-headed parrot	*Poicephalus cryptoxanthus*	LC	Stable	Southern and East Africa	[102, 103]
Red-fronted parrot	*Poicephalus gulielmi*	LC	Decreasing	West, Central and East Africa	[104, 105]
Cape parrot	Poicephalus robustus*	LC	Decreasing	West, Central, East and Southern Africa	[25, 80]
Rüppell’s parrot	*Poicephalus rueppellii*	LC	Decreasing	Southern and Central Africa	[31, 104]
Red-bellied parrot	*Poicephalus rufiventris*	LC	Stable	East Africa	[31, 104]
Senegal parrot	*Poicephalus senegalus*	LC	Stable	West Africa	[79, 104]
Regent parrot	Polytelis anthopeplus*	LC	Decreasing	Oceania	[80, 106]
Palm cockatoo	*Probosciger aterrimus*	LC	Decreasing	Oceania and South East Asia	[9, 75]
Red-rumped parrot	*Psephotus haematonotus*	LC	Increasing	Oceania	[95]
Red-breasted parakeet	*Psittacula alexandri*	NT	Decreasing	South East and South Central Asia	[80]
Echo parakeet	Psittacula echo*	EN	Increasing	East Africa	[30]
Alexandrine parakeet	*Psittacula eupatria*	NT	Decreasing	South East and South Central Asia	[36, 58]
Rose-ringed parakeet	Psittacula krameri*	LC	Increasing	West, Central, East Africa; South Central Asia	[39, 107]
Edwards’ fig-parrot	*Psittaculirostris edwardsii*	LC	Stable	Oceania	[80]
African grey parrot	*Psittacus erithacus*	VU	Decreasing	West, Central and East Africa	[19, 108]
Timneh parrot	*Psittacus timneh*	VU	Decreasing	West Africa	[20, 58]
Scaly-breasted lorikeet	*Trichoglossus chlorolepidotus*	LC	Stable	Oceania	[80]
Olive-headed lorikeet	*Trichoglossus euteles*	LC	Stable	South East Asia	[80]
Scarlet-breasted lorikeet	*Trichoglossus forsteni*	NT	Decreasing	South East Asia	[80]
Rainbow lorikeet	Trichoglossus haematodus*	LC	Decreasing	Oceania and South East Asia	[48, 84]
Deplanche’s rainbow lorikeet	Trichoglossus haematodus deplanchii*	Not assessed		Oceania	[95]
Red-collared lorikeet	*Trichoglossus rubritorquis*	LC	Decreasing	Oceania	[29, 65]
Yellow-tailed black-cockatoo	*Zanda funerea*	LC	Stable	Oceania	[109]

The summarised captive and wild population BFDV prevalence estimates are reported in Table 2. Prevalence estimates have been provided for nine national captive populations globally, comprising four from Europe (two of which were for Poland), two from Oceania, two from East Asia and one from Central America. These estimates vary in their scope, from describing prevalence in a subset of species (e.g., parakeets, [42]) to estimating BFDV prevalence across entire national captive populations (e.g. [43]). Among wild populations, seven of eight publications reporting prevalence estimates are from the Oceania region, with four from New Zealand alone. *Cacatua galerita* populations in Australia were estimated to have a viral prevalence of between 10 and 20 % [44], slightly below the minimum estimate provided for populations in New Zealand two decades later [45]. The lower limits of the 95 % confidence interval surrounding BFDV prevalence in wild *Platycercus eximius* populations in New Zealand provided by two separate research groups, five years apart, are comparable [45, 46]. However, the upper limit varies from 20.4 % to more than double, at 42.3 %. Similarly, the two estimates for *Cyanoramphus novaezalandiae* populations differ greatly from one another [46, 47], with the upper limit of the 2012 estimate approximately 12 % lower than the total estimate provided in 2009. The only estimates for African populations are from Mauritius, where the endemic parakeet population was screened annually throughout the duration of the study. From 2004 to 2009, the estimated total prevalence varied from 11 to 41 % [30].

**Table 2:** BFDV prevalence estimates and the screening tests used in publications from 1984 to 2015 for both wild and captive psittacine populations

Population location	Test prevalence	Methods used	Reference
**Captive**			
Germany	39.2 % from 32 captive breeding facilities	PCR	[110]
Australia	23 % (PCR)/66.7 % (HA) of samples submitted by veterinarians	PCR, HA, HI	[65]
Italy	8.05 % for entire national captive population	PCR	[43]
Taiwan	41.2 % of birds submitted by private owners	PCR	[111]
New Zealand	<7 % cumulative parakeet species	PCR, Histology	[42]
Poland	25.3 % for entire national captive population; 22.12 % - small aviaries; 25.23 % - medium aviaries; 25.99 % - large aviaries	PCR	[112]
Costa Rica	19.7 % for entire national captive population	PCR	[59]
Japan	31.3 % of imported birds for breeding	PCR	[58]
Poland	20.6 % across 50 captive breeding facilities	PCR, Whole-genome sequencing	[36]
**Wild**			
Australia	*Cacatua galerita* - 10 - 20 % (200 - 1000 individuals) over 4 years	Histology	[44]
New Zealand	*Platycercus eximius* - 8.6-20.4 %, *Cacatua galerita* - 22-33 %	PCR, Histology	[45]
New Zealand	4–7 % across all native species	PCR, Histology	[42]
New Zealand	*Cyanoramphus novaezalandiae* - 28 %	PCR	[47]
New Zealand	*Cyanoramphus novaezalandiae* - 10.5 % (95 % CI: 6.1 %–16.4 %); *Cyanoramphus auriceps* - 26.7 % (95 % CI 12.3 %–45.9 %); *Platycercus eximius* - 22.9 % (95 % CI 9.9 %–42.3 %)	PCR, Whole-genome sequencing	[46]
Mauritius	*Psittacula echo* - 2004/05 - 38 %; 2005/06 - 41 %; 2006/07 - 11 %; 2007/08 - 25 %; 2008/09 - 17 %	PCR	[30]
New Caledonia	*Trichoglossus haematodus deplanchii* - 25 % (11-45 %)	PCR, Whole-genome sequencing	[40]
Australia	*Platycercus elegans* - 45-50 %; *Platycercus elegans adelaidae* - 95-100 %; *Platycercus elegans flaveoulus* - 18-22 %, WS hybrids - 8-10 %	qPCR, HI	[54]

### Most frequently used laboratory methods

Of the 83 publications evaluated, 48.2 % (n = 40) of them used a single method for detecting BFDV, with standard PCR-based assays being the most frequently applied (42.5 %), followed by whole-genome sequencing (27.5 %) and histology (17.5 %), respectively.

**Fig. 3:**
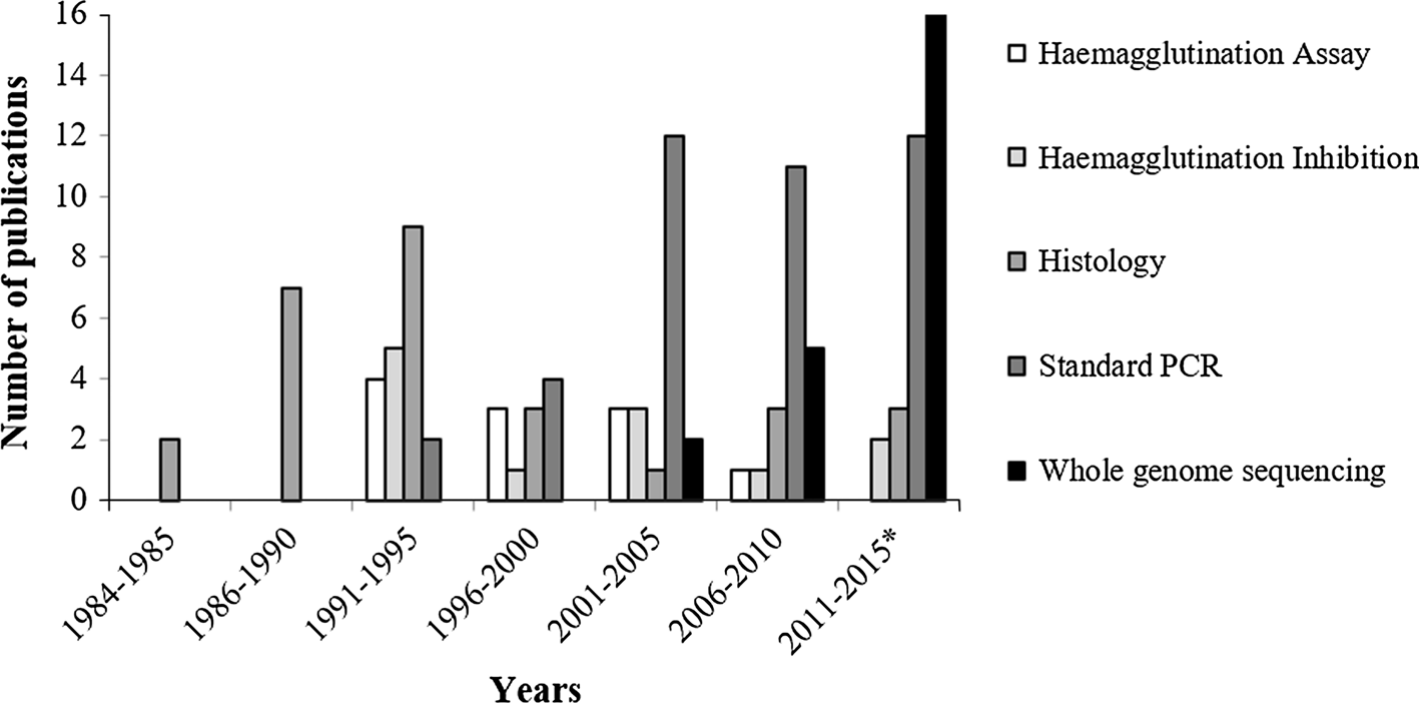
Changes in the frequency of use of the five most common screening and diagnostic methods used for detecting BFDV and PBFD between 1984 and July 2015

Histology using both light and scanning electron microscopy has been one of the most frequently and consistently used methods from 1984 to present. Of the 14 methods available for screening and diagnostics, histology has been used at least once in combination with all but quantitative (or real-time) polymerase chain reaction (qPCR), blocking ELISA and duplex shuttle PCR. An ELISA test was first developed for screening in the mid-1980s [18], but no BFDV screening-based publications used this method until more than two decades later (Table 3), after which it was never used again. Similarly, the duplex shuttle PCR method has been used only once. Both HA and HI were used on 12 occasions since their first application in 1991 (Table 3). However, HA was not used at all in the most recent (2011 to July 2015) publication period (Fig. 3).

**Table 3:** A summary of all methods used in screening for BFDV in wild and captive psittacine populations, a count of how many published studies in which each has been used and example publications for where each has been applied

Method	Description	Times used	Example references
Blocking ELISA	A blocking ELISA is a method used to immobilize biomolecules, primarily proteins, to a plate via passive or covalent interactions, minimising nonspecific binding to the surface by saturating unoccupied binding sites with a blocking reagent	1	[109]
DNA *in situ* hybridization	DNA *in situ* hybridization is a technique used in the localisation of specific nucleic acid targets within fixed tissues and cells using an oligonucleotide probe before microscopically visualizing the results	4	[69, 81]
Dot-blot DNA hybridization	Dot blot hybridization is a technique used to determine the abundance of certain DNA in an extraction dotted onto a membrane through hybridization with universal and specific oligonucleotide probes	2	[20, 21]
Duplex shuttle PCR	Duplex shuttle PCR is a process that allows the co-amplification of separate regions of a gene in a single PCR reaction using different pairs of primers in the same reaction mixture	1	[51]
Endocrinological response	Endocrinological response is a method used to challenge the host immune system with a hormone that encourages the production and release of a stress hormone to evaluate whether any differences exist between healthy and infected individuals	1	[87]
Haemagglutination assay	Haemagglutination assay (HA) is a method used to quantify the amount of virus attached to molecules on the surface of host red blood cells in a series of dilutions of a viral suspension	12	[26, 113]
Haemagglutination inhibition	A modified version of the HA where a standard amount of virus and host blood cells are used with the addition of a serially diluted antiserum to determine which concentration inhibits agglutination of the cells	12	[9, 65]
Haematology	Haematology is the study of the morphology and physiology of blood and, in this context, relates to the diagnosis and monitoring of pathogens present in the blood stream	3	[87, 89]
Histology	Histology is the microscopic examination of stained tissues and is applied in the screening for BFDV to determine if viral inclusion bodies are present. Techniques include light and electron microscopy	28	[57, 114]
Immunohistochemical tests	Immunohistochemistry (IHC) is a technique used to observe the physical characteristics of antibodies and their concentration and distribution within host tissue. In screening for BFDV, specimens are stained using the avidin-biotin complex (ABC) immunoperoxidase technique and then exposed to a primary antibody	5	[19, 91]
Quantitative (real-time) PCR	Quantitative (or real-time) polymerase chain reaction (qPCR) is a technique used to both amplify and quantify target DNA through the use of either nonspecific fluorescent dyes that intercalate with double-stranded DNA or a sequence-specific fluorescent probe that hybridizes with the target	6	[37, 54]
Standard PCR	Polymerase chain reaction (PCR) is a technology used to amplify a piece of DNA across several orders of magnitude through a process of thermal cycling in combination with oligonucleotide probes synthesised to bind to the target region and a DNA polymerase enzyme	41	[48, 102]
Virus purification	Virus purification allows the careful study of viral synthesis within cells by combining ultracentrifugation, adsorption chromatography, electrophoresis, and partition in liquid phases to separate virions from incomplete protein fragments and cell debris	3	[26, 75]
Whole-genome sequencing	Whole-genome sequencing is a laboratory process that determines the complete DNA sequence of an organism’s genome at a single time and can be used for multiple tissue types and when only very small quantities of a full DNA copy are present	23	[115, 116]

The standard PCR-based assay has been the most frequently used screening method, applied in 49.4 % of reported studies between 1984 and July 2015 (Table 3). The method was initially applied in only two of 11 studies published in the period 1991-1995 but was consistently the most used between 1996 and 2010. Of the 35 publications that used standard PCR from 2000 onwards, 24 used the protocol and/or oligonucleotide primers developed by Ypelaar *et al.* [48]. The application of both PCR and whole-genome sequencing is considerably higher than any other mixed-method approach. These were used together in 12 studies, in nine of which they were the only methods used. In the period from 2011 to July 2015, applications of whole-genome sequencing exceeded those of standard PCR for BFDV screening (Fig. 3) and were used in 52.3 % of publications since its first application in 2004. Rolling-circle amplification using Phi29 DNA polymerase [49] was first used for whole-genome sequencing of BFDV in 2005 and has been applied in 47.8 % of studies using this technique. Subsequently, the methods as described by Shepherd *et al.* [50] have been applied across all studies using Phi29 DNA polymerase for BFDV whole-genome amplification since its publication.

### Tissue types used for screening

A total of 13 tissue types have been used for BFDV screening since 1984: beak, blood, bone marrow, cloacal swabs, crop samples, embryonated and non-embryonated eggs, faeces, feather dust, feathers, muscle tissue, skin and viscera. All tissue types, aside from beak, have been used for screening on at least one occasion in captive populations, with feathers used the most frequently (34.2 %), followed by blood (32.5 %) and viscera (13.7 %). Conversely, only six tissue types have been used in the screening of wild populations. As with captive populations, blood (41.2 %) and feathers (37.3 %) were the most commonly used source for samples, with viscera studied 9.8 % of the time and beak used on only one occasion.

### Descriptions of clinical signs

Basic visual body condition assessments were mentioned in 36 of the 83 publications and ranged from a brief statement of the presence or absence of feather disorder [20, 51] to more in-depth observations regarding overall body condition [39, 52]. More-thorough scoring systems for the classification of clinical symptoms were applied in eight studies. The most descriptive of these systems was by Regnard *et al.* [37], consisting of six different clinical symptoms, with each broken down into five different scores of overall physical condition, and these scores were then compared to individual viral load. Other scales, such as that applied by Ritchie *et al.* [9, 17], descriptively scored only clinical feather and beak lesions.

### Field methods used to obtain wild specimens

Only 16 of the 38 studies reporting BFDV incidence in wild birds discussed the field methods used to obtain their specimens. The most frequently used method was mist netting, reported in 11 of the 16 publications (e.g. [37, 40, 46]). The second most preferred method was trapping, either whilst individuals were in nests [44, 53] or with walk-in traps [53, 54]. Other studies were undertaken on specimens gathered opportunistically from mortality cases and individuals brought in for health checks [42].

## Discussion

### Patterns in global PBFD and BFDV research

Interest in the screening for, spread and impact of BFDV and PBFD globally has steadily increased over the last three decades, with a particular focus on wild populations in the last five years. Over the course of this period, the focus in research has shifted from basic descriptions of patterns of presence or prevalence in populations towards studies investigating the processes of viral recombination, evolution and phylogenetics (e.g. [36, 55]), the causes of outbreaks in wild populations (e.g. [28, 40]), and the implications for improving the management of captive and wild populations. However, despite the burgeoning interest in assessing incidence in wild populations, some conspicuous research gaps are apparent, which future research should aim to fill. Oceania is undoubtedly the geographical region that has received the most research attention regarding the incidence of BFDV in both wild and captive populations. This geographical bias may partly be due to evolutionary studies suggesting the virus likely originated from this region, as well as the recognition of PBFD as a disease of concern to the recovery of endemic parrot populations there and a key threat to biodiversity [32, 56]. In contrast, there has been very little published research on BFDV in proximate geographical regions of high parrot diversity such as Southeast and Southern Asia.

This bias in research attention to some extent likely reflects publication bias against negative results. The authors are aware of several screening studies in which the virus was not detected but the results of these studies have not been published and hence have not been included in this review. There is a need to make the results of such screening initiatives publicly available for further scrutiny, especially in light of the evidence that some species, such as cockatiels (*Nymphicus hollandicus*), may be less susceptible to BFDV infection [57]. Many aviculturists routinely test translocated birds for BFDV, but there is little incentive to publish the results of such tests; indeed, there may even be disincentives to publish positive results among commercial breeders. Approaches to gathering test results that preserve anonymity may improve the availability of data.

Given that *Cacatua* was the genus from which PBFD was first described, 11 species of which have proven to be susceptible to BFDV infection, to date, there has been very little research on BFDV/PBFD in areas of Southeast Asia to which many of these species are native. The virus has been found in specimens from both wild and captive populations in Indonesia, a country that contains many psittacine breeding farms [58] and is heavily exploited for both the legal and illegal trapping and export of companion birds for the pet trade [41]. Equally, with high levels of parrot endemicity in South and Central America, it is surprising that no studies have been published on BFDV or PBFD incidence in wild populations. Only two studies have been conducted on captive individuals originating from these geographical regions: one from Costa Rica [59] and another that included specimens of Guyanese origin [58]. Whilst one study from Brazil did not specify whether the individuals studied were of captive or wild origin and were therefore not included in Fig. 2 [60], this anomaly makes little difference to the overall picture. Similarly, most of the African continent is data deficient, with no BFDV studies published on wild populations north of Zambia [61] or from any of the Indian Ocean islands other than Mauritius. The captive studies have been slightly more inclusive, with specimens from Cameroon and the Ivory Coast, but they were not conducted within the country of origin and therefore provided little information on the state of captive flocks locally. Also, as the specimens from captive birds originating from these nations tested positive for BFDV [43] it would be beneficial to investigate wild populations further for the occurrence of any spillover from the aviculture industry.

Notably, one species that requires further research focus is *Psittacula krameri*, the most introduced parrot globally with breeding populations in approximately 35 countries across five continents [62]. No BFDV screening has been conducted on any of the wild populations of *P. krameri* across its extensive native range in Africa and Asia. However, feral populations within its invasive range and captive individuals have tested positive for BFDV [30, 36, 39]. It is therefore highly likely that the virus is present in wild flocks, which may act as a reservoir with potential spillover into other sympatric vulnerable psittacine species.

### Advances in methods

The variety of optimised diagnostic tests and technologies available for BFDV screening have increased and improved substantially since its first scientific assessment. Whole-genome sequencing has become a particularly prominent tool in recent years due to the small size of the BFDV genome, reduced costs of this technique, and the availability of comparable sequence data through collective resources such as GenBank. The application of rolling-circle amplification has greatly simplified and improved whole-genome amplification of circoviruses for further analysis using microarrays [63] and next-generation sequencing techniques [64], particularly when variant sequences are present. Other methods, such as blocking ELISAs, duplex shuttle PCRs, and dot-blot DNA hybridization have been used once or twice but were not as effective as other methods available or in common use at the time. Unlike the ELISA, the HI assay, currently the leading assay for anti-BFDV antibody detection, does not require a secondary antibody and is widely suitable for detection for a large proportion of psittacine species [65].

Standardisation of approaches to basic viral screening would improve both accuracy and repeatability and allow more reliable modelling, extrapolation and population prevalence estimates that are comparable between countries, species or breeding facilities. These data could facilitate research into important aspects of the epidemiology of PBFD, such as pathogenesis in wild populations, virulence and transmission. In addition, standardised approaches and improved detection accuracy would support conservation practitioners and managers of captive breeding facilities. For example, increased confidence in diagnostic tests would assist decisions over the translocation of birds in species recovery and reintroduction programmes and might help to avoid the introduction of infected individuals into disease-free captive collections. Whilst steps have recently been taken to improve the standard PCR protocol by quantifying DNA extraction concentrations prior to screening [53], an assessment of detection accuracy at variable DNA concentrations and how this impacts the repeatability of a result is still lacking in the literature.

Quantitative (real-time) PCR techniques are now being more regularly applied to determine individual viral load [37, 53, 66], as probe-based assays are able to detect viral DNA at much lower concentrations than approaches that rely on detection by the naked eye when visualizing a gel. However, the reagents and equipment required for screening using standard PCR are currently substantially less expensive than those used for probe-based assays and are thus likely to have continued widespread use for the purpose of general BFDV screening.

### Tissue types used for screening

Extracted DNA samples can vary greatly in yield depending on the type and amount of tissue used. For example, feathers typically produce very low genomic DNA yields, particularly when extracted from those that are cut off from the blood supply once fully grown [67], only representing viral incidence during the initial growth phase. Concentrations can considerably affect the sensitivity of PCR assay [65], as the amount of viral DNA obtained from any sample will be dependent on the infection level within the host at the time of sampling [5, 68], making higher DNA yields preferable to increase the probability of detection. A number of studies have proven that there are inconsistencies in detection of BFDV between tissue types [53, 65, 69, 70]. Feathers have been found to test positive for BFDV in the absence of clinical signs [70], in cases in which no HI antibody was detectable [65] and when an individual’s blood or tissue tested negative [53].

Whilst samples from wild populations may be easier and require less veterinary expertise to obtain through non-invasive techniques, such as the collection of feathers, there is a higher risk of cross-contamination between samples [71] and thus may increase the proportion of false-positives when screening. Also, as a primary symptom of PBFD is feather loss, the collection of dropped feathers (for example from a roost site) may further bias the estimated proportion of infected individuals. Therefore, as with the variation in diagnostic methods, it would be valuable to standardise a protocol each for blood and feathers (the two most commonly screened tissue types) for widespread use between managers of both wild and captive populations. As the screening of muscle tissue and blood have been found to provide highly comparable results with standard and qPCR techniques [53], a standardised blood screening protocol including host DNA quantification and an estimate of false-negative error could therefore also be extended to use with other internal tissues such as muscle or viscera.

### Reporting of body condition

Both the body condition of screened individuals and the techniques used to capture wild birds have been inconsistently reported in the literature. As it has been shown that some individuals can remain asymptomatic despite testing positive for BFDV [10], it is difficult to determine whether body condition assessments are of value in informing management guidelines. However, overall physical condition has been found to correlate with viral load in Cape parrots [37], and consequently, it may be of value to implement a robust and standardised scale of clinical signs as a primary means of assessment in the field. This finding will need to be tested in a number of other parrot species to determine its repeatability across the family *Psittacidae* before further reliance can be placed on this as a means of indirectly inferring host prevalence without a diagnostic test.

### Standardisation of field techniques

The under-reporting and failure to standardise techniques used in the field limits the potential to make direct comparisons between studies. While efforts should be made to standardise approaches wherever possible, it is important to recognise that the practicalities of sampling each study system may limit the approaches that can be used. However, it should be taken into consideration that if a large number of BFDV- or PBFD-positive birds are captured, this may be due to a bias in the method of capture towards weaker or diseased individuals. Additionally, mist nets and traps, the most frequently used field techniques used to catch wild birds, may facilitate the horizontal spread of infection between individuals if equipment is not adequately cleaned between uses. BFDV has been found to be highly environmentally persistent [28], and conservation managers should therefore be aware of the risks of increased transmission when a thorough cleaning regime is not implemented.

### Applications

The application of screening and diagnostic tests for BFDV has developed from trying to understand the structure of the virus, how it is transmitted between individuals, and the nature of the disease to assessing what incidence and prevalence means for conservation management and interrogating evolutionary relationships between strains. These methodological developments have proven to be particularly valuable when considering translocation and reintroduction programmes for wild populations [72, 73], highlighted by the loss of a new founder population of endangered *Psittaculaecho* to PBFD in 2005 [35, 74].

Initially, the virus was thought to be limited in its diversity [75], and early attempts to produce a protective vaccine indicated that this approach could be useful for preventing PBFD [18, 76, 77]. Little attention has been given to this in recent years despite it being listed as a high priority in a threat abatement plan for PBFD in Australian birds [56]. Instead researchers and practitioners have focused on closer monitoring and management of infection and disease; trying to avoid spillover into vulnerable species [28, 40].

Our review highlights the need for greater research focus on PBFD and BFDV in wild parrot populations, particularly when taking into consideration the intrinsic connection between the trade in companion birds and the spread of novel BFDV strains into the wild [34]. Understandably, the application of the term “EID” when referring to any pathogen needs to be carefully considered in light of its endemicity and virulence in the affected host species. Given the number of species, subspecies and global regions now affected by BFDV, and the recent increase in its reported occurrence in threatened wild parrot populations, it may now be appropriate to consider this pathogen to be an EID. It is clear, however, that there are still many opportunities to study the impact of infection and disease in captive and wild parrot populations within their countries of origin across the Americas, Africa and Asia. Many parrot species have declining populations and exist within highly fragmented and degraded habitats [41], and consequently, it would be of great value in the future conservation of wild populations to determine how the spread of infectious disease further affects survival or persistence. Only a few total prevalence estimates exist for captive and wild populations. These provide valuable information for geographical and cross-species comparisons that, in some cases, could be relatively easily reported by modelling existing data on the proportion of infected individuals or samples obtained when screening. The progression and refinement of the screening and diagnostic tools currently available for the study of BFDV would allow a broader application of results in management strategies and in disease transmission prevention protocols. The standardisation of sampling methodologies and diagnostic assays would be an important step towards improved understanding of the epidemiology of PBFD and BFDV and management of both captive and wild populations.
